# seqme: a Python library for evaluating biological sequence design from generative models

**DOI:** 10.1093/bioadv/vbag212

**Published:** 2026-08-07

**Authors:** Rasmus Møller-Larsen, Adam Izdebski, Jan Olszewski, Pankhil Gawade, Michal Kmicikiewicz, Wojciech Zarzecki, Ewa Szczurek

**Affiliations:** Institute of AI for Health, Helmholtz Munich, Neuherberg 85764, Germany; School of Computation, Information and Technology, Technical University of Munich, Garching bei München 85748, Germany; Institute of AI for Health, Helmholtz Munich, Neuherberg 85764, Germany; School of Computation, Information and Technology, Technical University of Munich, Garching bei München 85748, Germany; Faculty of Mathematics, Informatics and Mechanics, University of Warsaw, Warszawa 02-097, Poland; Institute of AI for Health, Helmholtz Munich, Neuherberg 85764, Germany; School of Computation, Information and Technology, Technical University of Munich, Garching bei München 85748, Germany; Institute of AI for Health, Helmholtz Munich, Neuherberg 85764, Germany; School of Computation, Information and Technology, Technical University of Munich, Garching bei München 85748, Germany; Faculty of Mathematics, Informatics and Mechanics, University of Warsaw, Warszawa 02-097, Poland; Faculty of Electronics and Information Technology, Warsaw University of Technology, Warszawa 00-665, Poland; Institute of AI for Health, Helmholtz Munich, Neuherberg 85764, Germany; Faculty of Mathematics, Informatics and Mechanics, University of Warsaw, Warszawa 02-097, Poland

## Abstract

**Summary:**

Recent advances in computational methods for designing biological sequences have sparked the development of metrics to evaluate these methods performance in terms of the fidelity of the designed sequences to a target distribution and their attainment of desired properties. However, a software library implementing these metrics was lacking. In this work we introduce seqme, a modular and highly extendable open-source Python library, containing model-agnostic metrics for evaluating computational methods for biological sequence design. seqme considers three groups of metrics: sequence-based, embedding-based, and property-based, and is applicable to a wide range of biological sequences: small molecules, DNA, ncRNA, mRNA, peptides and proteins. The library offers a number of embedding and property models for biological sequences, as well as diagnostics and visualization functions to inspect the results. seqme can be used to evaluate both one-shot generation and iterative optimization. We show the utility of seqme by performing an antimicrobial peptide benchmark and acquiring mRNA data.

**Availability and implementation:**

seqme is released at https://github.com/szczurek-lab/seqme under the BSD 3-Clause license.

## 1 Introduction

Biological sequences are composed of ordered chains—of nucleotides in DNA and RNA, amino acids in peptides and proteins, and atoms in small molecules. These sequences carry the information on structure, interactions, and function of the encoded molecules, making sequence modeling central to understanding and engineering biology (Anfinsen 1973, Kozak 1986). Recent years have witnessed a surge of generative AI models and algorithms for biological sequence design, including the development of novel compounds (Tang *et al.* 2024, Izdebski *et al.* 2025), peptides (Szymczak *et al.* 2025), and proteins (Kortemme 2024, Kmicikiewicz *et al.* 2025). Evaluating biological designs requires multiple complementary criteria, such as fidelity to a reference dataset’s distribution, novelty, diversity, and optimization of desired properties. Overlooking these aspects when evaluating computational methods, in particular generative AI models, can result in failures to generate sequences that are both high quality and functionally useful (Theis *et al.* 2016). Therefore, a plethora of evaluation metrics have been proposed to measure performance and identify failure modes of generative models (Manduchi *et al.* 2025). However, to date, no software library has been developed that unifies these efforts and is dedicated specifically to evaluating biological sequence design.

To address this gap, we introduce seqme—the first library for end-to-end evaluation of generative AI and computational algorithms for *de novo* biological sequence design. The library provides access to a collection of metrics, embedding models and property predictors, while remaining highly extensible. It supports major types of biological sequences, including small molecules, DNA, ncRNA, mRNA, peptides, and proteins. In addition, seqme enables assessment of single-shot design and iterative sequence discovery pipelines. By offering a unified and reproducible framework, the library establishes a practical *foundation* for fair comparison and accelerated progress in biological sequence design.

## 2 Metrics

seqme offers metrics for measuring *distribution-* and *property*-based quality of sequence designs, i.e. the degree to which the sequences resemble a biological reference distribution and to which they satisfy pre-specified target properties. Depending on whether the metrics operate directly on raw sequences, on their learned embeddings, or on derived properties, we further categorize them into three classes: sequence-, embedding-, and property-based metrics, respectively. The implemented metrics are summarized in Table 1. Definitions and the biological domains where each metric can be applied are described in Supplementary Text, available as supplementary data at *Bioinformatics Advances* online.

**Table 1:** Metrics implemented in seqme.^a^

	Metric	Description
Sequence	Novelty (↑) (D)	Fraction of sequences not in the reference set.
	Uniqueness (↑) (D)	Fraction of unique sequences.
	Diversity (↑) (D)	Normalized Levenshtein distances within or between sequences.
	N-gram Jaccard Similarity (↑/↓) (D)	Jaccard similarity between sequences and reference set’s n-gram set.
Embedding	Fréchet Biological Distance (FBD) (Heusel *et al.* 2018) (↓) (D)	2-Wasserstein distance between two multivariate Gaussian distributions fitted to the embeddings of the sequences and reference set.
	Maximum Mean Discrepancy (MMD) (↓) (D)	MMD between sequences and references embeddings using a Gaussian radial basis function kernel (Jayasumana *et al.* 2024) or a rational quadratic kernel (Bińkowski *et al.* 2021).
	Precision (Kynkäänniemi *et al.* 2019) (↑) (D)	Fraction of sequences that fall inside the support of the reference set.
	Recall (Kynkäänniemi *et al.* 2019) (↑) (D)	Fraction of references that fall inside the support of the sequences.
	Authenticity (AuthPct) (Alaa *et al.* 2022) (↑) (D)	Fraction of sequences whose nearest training neighbor is closer to some other training sample than to the sequence.
	Fourier-based Kernel Entropy Approximation (FKEA) (Ospanov *et al.* 2024) (↑) (D)	Vendi and Rényi Kernel Entropy (RKE) score (Friedman and Dieng 2023) approximation via random Fourier features for diversity estimation.
Property	Property moment (ID) (↑/↓) (P)	Mean and standard deviation of a single property across the sequences.
	Hit-rate (↑) (P)	Fraction of sequences satisfying a user-defined set of conditions.
	Hypervolume (↑) (P)	Hypervolume of two or more properties derived from the sequences. Computed either as the hypervolume indicator (Zitzler and Thiele 1999) or the convex-hull.
	Conformity score (Frey *et al.* 2024) (↑) (D)	Distributional similarity of the sequences properties and the references properties.
	KL-divergence (↓) (D)	Kullback–Leibler divergence between sequences and references for a single property.

a Arrows indicate whether a metric ought to be maximized (↑) or minimized (↓). (D) and (P) indicate *distribution-* or *property*-based sequence quality evaluation. For an up-to-date list, check https://seqme.readthedocs.io/en/stable/api/metrics_index.html.

### 2.1 Sequence-based metrics

seqme includes the metrics *novelty*, *diversity*, and *uniqueness*, which are commonly used to detect failure modes. There is a tradeoff between maximizing these metrics and optimizing user-defined properties. A typical failure mode is overfitting to a small set of sequences that satisfy the target properties at the expense of novelty, diversity, and uniqueness. Conversely, a random baseline can trivially maximize novelty and diversity without actually optimizing the desired properties, since the sequence space in biological domains is huge. Thus, these metrics are necessary for a comprehensive evaluation of biological sequence design methods.

### 2.2 Embedding-based metrics

seqme implements the metric *Fréchet Biological Distance* (FBD) (Heusel *et al.* 2018), which is commonly used to evaluate the distributional similarity between biological sequences designed by a model and those in a reference dataset (Preuer *et al.* 2018, Stark *et al.* 2024, Faltings *et al.* 2026). However, FBD is sensitive to both the number of sequences and their distribution in the embedding space. Therefore, seqme also includes *Maximum Mean Discrepancy* (MMD), which provides more stable estimates (Jayasumana *et al.* 2024). In addition, the library implements reference-free metrics such as *Vendi-* and *RKE-score* (Friedman and Dieng 2023, Ospanov *et al.* 2024) to evaluate diversity in the embedding space. None of the aforementioned metrics can identify memorization, i.e. near-sequence copying in the embedding space. To address this, seqme includes the *Authenticity* metric (Alaa *et al.* 2022). Finally, the library also implements *Improved Precision* and *Improved Recall* (Kynkäänniemi *et al.* 2019) to evaluate fidelity and diversity in the embedding space.

### 2.3 Embedding models

Recall that embedding-based metrics rely on embedding models that map sequences to fixed-length vector representations. For protein embeddings, seqme includes ESM-2 (Lin *et al.* 2022), for peptides it contains ESM-2 finetuned on peptide sequences and Hyformer trained on peptides (Izdebski *et al.* 2025), for ncRNA and mRNA, it uses RNA-FM (Chen *et al.* 2022), for DNA it includes GENA-LM (Fishman *et al.* 2025) and for small molecules Hyformer trained on small molecules (Table 1, available as supplementary data at *Bioinformatics Advances* online). The library also supports the use of alternative embedding models.

Selecting an appropriate embedding model is a non-trivial task as the embeddings must capture the biological domain of interest. To assist with this, seqme provides functionality for visualizing the embedding space using PCA, t-SNE, and UMAP 2D projections. Furthermore, seqme offers diagnostic tools, such as the k-nearest neighbor feature-alignment score and Spearman alignment score (Rissom *et al.* 2025), to evaluate how well embedding models align with discrete or continuous sequence properties of interest. See Supplementary Text, available as supplementary data at *Bioinformatics Advances* online, for more details.

### 2.4 Property-based metrics

seqme implements, among others, the metrics *Conformity Score* (Frey *et al.* 2024) and *Hit Rate*. The latter is commonly used in drug discovery pipelines to quantify the fraction of sequences that satisfy desired properties. In addition, seqme includes the multi-objective optimization metric *Hypervolume Indicator* (Zitzler and Thiele 1999), which computes the hypervolume of two or more properties across a set of sequences.

### 2.5 Property models

Property-based metrics assume access to property models. seqme provides functionality to compute several physico-chemical properties of molecules, peptides, and proteins, as well as models predicting whether a peptide exhibits antimicrobial activity (Table 1, available as supplementary data at *Bioinformatics Advances* online). Furthermore, seqme integrates ESMFold (Lin *et al.* 2022) and ESM-IF1 (Hsu *et al.* 2022), which are structure-aware models that predict a protein’s three-dimensional structure from its sequence and, conversely, infer the sequence from a given structure, respectively. Finally, the library contains models to predict DNA promoter regions and splice sites.

## 3 Additional functionalities of seqme

### 3.1 Fold functionality

Metrics, such as *Improved Precision* and *Improved Recall* (Kynkäänniemi *et al.* 2019), can become biased when there is a discrepancy in the number of designed sequences and reference sequences. Furthermore, for any evaluated metric, it is often desirable to estimate its standard deviation across multiple subsets of the designed sequences. To address these matters, we introduce *Fold*, which splits the sequences into *K* groups of equal size and computes the metric *K* times. This approach mitigates metric-specific sample-size biases, e.g. *FBD*, reduces the number of discarded sequences, and enables the estimation of standard error.

### 3.2 Support of iterative designs

Recall, seqme enables evaluation of a given tuple of sequences using a set of metrics of interest. This is convenient for one-shot design, where the given tuple of sequences is evaluated only once. On top of that, seqme also supports iterative evaluation workflows. This functionality enables the same metrics to be applied iteratively, e.g. to evaluate design across training steps of generative models, or throughout the iterative sequence refinement process in genetic algorithms and Bayesian optimization.

### 3.3 Visualizations

seqme provides several convenient functions for visualizing metric results. It can display a customizable table of metrics for all evaluated sequence groups and plot a parallel-coordinates chart, where each axis corresponds to one metric. For inspecting a single metric, seqme offers a barplot with optional error or deviation bars. For iterative sequence design, it provides functionality to plot metric trajectories across design iterations for multiple sequence designs. These visualizations make sequence-design performance easy to interpret and help reveal potential failure modes.

## 4 Implementation

seqme is implemented as a highly extendable Python library (v3.10 or greater) while maintaining a simple interface (see *Example usage* box). seqme offers capabilities for performance optimization by caching sequence representations, i.e. sequence embeddings and properties. Caching provides a substantial speed-up when using several metrics with the same sequence embeddings and the embeddings models are large, e.g. most language models. Caching reduces the time-complexity of computing *m* metrics with the same embedding model for *n* sequences from O(nm+k) to O(n+k), where $k=\sum_{i=1}^{m}c_{i}$ is the cost of computing the metrics from the embeddings. seqme also contains extensive documentation and tutorials on how to use the library and how to add new metrics and models. Notably, we show how to integrate models with dependency conflicts using seqme’s third-party interface.

Example usage 1 import seqme as sm

2

3 sequences = {

4  ”UniProt”: [“GFGD”,“DPWDWV”,“IEFFT”],

5  ”DBAASP”: [“PGLGFY”,“AAVLNA”,“LAHRYH”],

6}

7 cache = sm. Cache(

8  models = {

9   ”esm2”: sm.models.ESM2(“facebook/esm2_t6_8M_UR50D”)

10  }

11 )

12 metrics = [

13  sm.metrics.Diversity(),

14  sm.metrics.FBD(sequences[“UniProt”], cache.model(“esm2”))

15 ]

16 df = sm.evaluate(sequences, metrics)

17 sm.show(df)

## 5 Case studies

We present two distinct use cases of seqme. Extensive details are provided in the Supplementary Text, available as supplementary data at *Bioinformatics Advances* online.

### 5.1 Benchmarking antimicrobial peptide design models

In this case study, we evaluate the performance of five existing generative models for antimicrobial peptide (AMP) design. Based on the five metrics employed, we find that there is a trade-off in the generative model’s designs diversity, fidelity to natural peptides, and having antimicrobial properties. Indeed, no single model outperforms all others across the evaluated metrics, as shown by Pareto ranking the models’ calculated metrics using seqme.

### 5.2 Acquiring high-quality mRNA data

In this case study, the goal is to select a subset of mRNA sequences from a larger pool for subsequent wet-lab evaluation, subject to a fixed sequence budget. This is a common scenario when selecting designed sequences from generative or sequences mined from biological databases. To achieve this, we run a genetic algorithm to select a subset of mRNAs from a larger pool. The fitness of mRNA subsets is evaluated using the metrics *FKEA* and the stability. By running a genetic algorithm primarily composed of functionality provided by seqme, we obtain more informative training data than we would by selecting sequences at random.

## 6 Discussion

seqme aims to ease the task of evaluating biological sequence design methods by providing metrics, as well as embedding and property models. While the library is already applicable to multiple biological sequence types, it is easily extendable to more types by adding type-specific embedding and property models.

We encourage users to use multiple metrics provided by seqme, as each metric offers a different perspective. This is of particular importance as each evaluation metric has limitations and can fail to detect a failure mode in a generative AI model or algorithm used for the design (Räisä *et al.* 2025). Only a well-chosen set of metrics will yield a robust evaluation.

We envision seqme will accelerate *de novo* drug discovery by providing the foundation for robust model benchmarking, and offering more comprehensive tools for defining stopping criteria in machine-learning training loops. Ultimately, we expect that libraries like seqme will enable practical applications of generative AI and other sequence design methods, and facilitate the translation of computational advances into biology and medicine.

## Supplementary Material

vbag212_Supplementary_Data

## Data Availability

seqme is installable using pip install seqme through PyPI. Library code is available at https://github.com/szczurek-lab/seqme. Code to reproduce the case studies is available at https://github.com/szczurek-lab/seqme-reproducibility. Documentation and tutorials are available at https://seqme.readthedocs.io. Third-party models are available at https://github.com/szczurek-lab/seqme-thirdparty. Library dependencies and their licenses are found in the Supplementary Text, available as supplementary data at *Bioinformatics Advances* online.
