# Supplementary material for: seqme: a Python library for evaluating biological sequence design from generative models
Source: Bioinform Adv. 2026 Aug 7;6(1):vbag212. doi: 10.1093/bioadv/vbag212 (PMC13499220; doi:10.1093/bioadv/vbag212)
Supplement: Supplementary file 1 [file vbag212_supplementary_data.pdf]

`seqme`: a Python library for evaluating biological sequence design from generative models Supplementary Material

Rasmus Møller-Larsen, Adam Izdebski, Jan Olszewski, Pankhil Gawade, Michal Kmicikiewicz, Wojciech Zarzecki, Ewa Szczurek

Contents

1 Supplementary Text **2**

1.1 Definitions . . . 2

1.1.1 Data . . . 2

1.1.2 Representation functions . . . 2

1.1.3 Metric . . . 2

1.2 Metrics . . . 2

1.2.1 Metrics from string representation . . . 3

1.2.2 Metrics from fixed-size embeddings . . . 3

1.2.3 Metrics from sequence properties . . . 5

1.3 Limitations of the metrics . . . 6

1.4 Diagnostics . . . 7

1.4.1 k-NN feature-alignment score . . . 7

1.4.2 Spearman alignment score . . . 7

1.5 Case studies . . . 7

1.5.1 Case study 1: Benchmarking antimicrobial peptide design models . . . 7

1.5.2 Case study 2: Acquiring high-quality mRNA data . . . 12

1.6 Comparison to existing frameworks . . . 13

1.7 Dependencies and licence . . . 15

2 Supplementary Tables **16**

1 Supplementary Text

1.1 Definitions

1.1.1 Data

In the definitions of metrics measuring performance of generative models, we will consistently refer to the following three objects:

1. $\mathcal{G}$ – set of generated outputs from the model.
2. $\mathcal{R}$ – set of reference data, which usually comes from the model's training or evaluation dataset.
3. φ – representation function, which usually decodes data to sequence strings or embedding vectors.

We assume that both the reference and generated sets share a common domain $\mathcal{D} \supset \mathcal{R}, \mathcal{G}$.

It is worth mentioning that we consider the elements $x, y \in \mathcal{G}$ different as long as they were generated by two distinct calls to the generative model, even if their string representations are identical

$$\varphi_{\text{string}}(x) = \varphi_{\text{string}}(y) \not\Longrightarrow x = y.$$

The same holds for elements of the reference set $\mathcal{R}$.

1.1.2 Representation functions

We distinguish between three categories of representation functions φ:

1. **String representation** which for each element assigns its decoded sequence. The sequence is a list of n letters coming from a finite alphabet $\mathcal{A}$. In the case of peptide sequences, $|\mathcal{A}| = 20$ is the alphabet of canonical amino acids. Formally
$$\varphi_{\text{string}} : \mathcal{D} \to \bigcup_{n=1}^{\infty} \mathcal{A}^n, \text{ where } \mathcal{A}^n = \{(a_1, a_2, \ldots, a_n) : a_i \in \mathcal{A}\}.$$
2. **Fixed-size embeddings** which employ some embedding model $\mathrm{E} : \varphi_{\text{string}}(\mathcal{D}) \to \mathbb{R}^d$, usually a pretrained deep learning model, to map the variable-length sequences to a fixed d-dimensional Euclidean space. Formally
$$\varphi_{\mathrm{E}} : \mathcal{D} \to \mathbb{R}^d, \text{ where } \varphi_{\mathrm{E}}(x) = \mathrm{E}(\varphi_{\text{string}}(x)).$$
3. **Sequence property** in which for each function $\mathrm{P} : \varphi_{\text{string}}(\mathcal{D}) \to Y$ assigning some property to a sequence, we define
$$\varphi_{\mathrm{P}} : \mathcal{D} \to Y, \text{ where } \varphi_{\mathrm{P}}(x) = \mathrm{P}(\varphi_{\text{string}}(x)).$$
Assumptions on the codomain Y will be introduced separately for each metric definition.

For any representation function $\varphi : \mathcal{D} \to Y$ and set $X \subset \mathcal{D}$ we define $\varphi(X)$ to be the image set $\{\varphi(x) : x \in X\}$.

1.1.3 Metric

A metric is a function m of generated data $\mathcal{G}$, which scores its quality

$$m : \mathcal{P}(\mathcal{D}) \to \mathbb{R}, \text{ where } \mathcal{P}(\mathcal{D}) = \{\mathcal{G} : \mathcal{G} \subset \mathcal{D}\}.$$

Optimizing the metric is desired, and the optimal set $\mathcal{G}^*$ is either minimizer $m(\mathcal{G}^*) = \inf_{\mathcal{G}}\{m(\mathcal{G})\}$ or maximizer $m(\mathcal{G}^*) = \sup_{\mathcal{G}}\{m(\mathcal{G})\}$ of the metric.

1.2 Metrics

In this section, we define metrics to evaluate generated data $\mathcal{G}$ based on reference data set $\mathcal{R}$ and representation function φ. All metrics are implemented in the `seqme` library. The metrics naturally split into three categories depending on the employed function φ to represent the data.

1.2.1 Metrics from string representation

In this group, metrics use the representation function φ_{string} which assigns a decoded sequence to each element $x \in \mathcal{D}$. We denote these metrics as sequence-based metrics.

Novelty is the fraction of generated data not included in the reference set

$$\text{Novelty}_{\mathcal{R}}(\mathcal{G}) = \frac{|\mathcal{G} \setminus \mathcal{R}|}{|\mathcal{G}|}. \tag{1}$$

Uniqueness is the fraction of unique sequences in the generated data

$$\text{Uniqueness}(\mathcal{G}) = \frac{|\varphi_{\text{string}}(\mathcal{G})|}{|\mathcal{G}|}. \tag{2}$$

Diversity measures the average pairwise dissimilarity between sequences in $\mathcal{G}$

$$\text{Diversity}(\mathcal{G}) = \frac{1}{|\mathcal{G}|} \sum_{x \in \mathcal{G}} \frac{1}{|\mathcal{G}|} \sum_{y \in \mathcal{G}} \overline{\text{lev}}(\varphi_{\text{string}}(x), \varphi_{\text{string}}(y)), \tag{3}$$

where $\overline{\text{lev}}(a, b)$ is the Levenshtein distance between strings normalized by the maximum string length

$$\overline{\text{lev}}(a, b) = \frac{\text{lev}(a, b)}{\max(\text{len}(a), \text{len}(b))}.$$

Because the cost of computing the Diversity metric grows quadratically in the size of the generated set $O(|\mathcal{G}|^2)$, we approximate the inner sum by sampling a random subset of k elements from $\mathcal{G}$ which does not contain x

$$\mathcal{G}_k(x) \sim \text{Unif}[\{\mathcal{G}' \subset \mathcal{G} \setminus \{x\} : |\mathcal{G}'| = k\}].$$

Therefore we get a k-approximation of Diversity

$$\text{Diversity}_k(\mathcal{G}) = \frac{1}{|\mathcal{G}|} \sum_{x \in \mathcal{G}} \frac{1}{k} \sum_{y \in \mathcal{G}_k(x)} \overline{\text{lev}}(\varphi_{\text{string}}(x), \varphi_{\text{string}}(y)). \tag{4}$$

The cost of computing Diversity_k is $O(nk)$. Note that $\text{Diversity}(\mathcal{G}) = \text{Diversity}_{|\mathcal{G}|-1}(\mathcal{G})$.

N-gram Jaccard similarity computes the average intersection over union for N-grams of generated data $\mathcal{G}$ and aggregated N-grams of reference data $\mathcal{R}$

$$\text{Jaccard}_{N,\mathcal{R}}(\mathcal{G}) = \frac{1}{|\mathcal{G}|} \sum_{x \in \mathcal{G}} \frac{\text{Ngram}(\varphi_{\text{string}}(x)) \cap \overline{\text{Ngram}}(\varphi_{\text{string}}(\mathcal{R}))}{\text{Ngram}(\varphi_{\text{string}}(x)) \cup \overline{\text{Ngram}}(\varphi_{\text{string}}(\mathcal{R}))}, \tag{5}$$

where the Ngram of a string is a set of its N-element substrings. Formally, for $s = \varphi_{\text{string}}(x)$ we define

$$\text{Ngram(s)} = \{s_{i:i+N} : 0 \le i \le \text{len}(s) - N\},$$

and for a set of strings $R = \varphi_{\text{string}}(\mathcal{R})$ the aggregated $\overline{\text{Ngram}}$ is defined as

$$\overline{\text{Ngram}}(R) = \bigcup_{s \in R} \text{Ngram(s)}.$$

1.2.2 Metrics from fixed-size embeddings

In this group, metrics use representation functions φ_{E} that assign a fixed-size d-dimensional embedding vector to each element $x \in \mathcal{D}$. We denote these metrics as embedding-based metrics. While generally less interpretable than sequence-based and property-based metrics, embedding-based metrics offer a key advantage: they leverage pretrained models that generalize across different sequence categories within the same domain, removing the need to train a new model from scratch, which can be time-consuming, computationally demanding, and often require more than a single reference dataset.

Fréchet Biological Distance (FBD) is defined identically to the *Fréchet Inception Distance* (FID) (Heusel et al., 2018), but replaces the Inception network with any biologically relevant embedding model E.

Assume that the embeddings of the generated data and the embeddings of the reference data follow a multivariate Gaussian distribution

$$\varphi_{\mathrm{E}}(\mathcal{G}) \sim \mathcal{N}(\mu_{\mathcal{G}}, \Sigma_{\mathcal{G}}),$$
$$\varphi_{\mathrm{E}}(\mathcal{R}) \sim \mathcal{N}(\mu_{\mathcal{R}}, \Sigma_{\mathcal{R}}).$$

The FBD between the generated data embeddings and the reference data embeddings is defined as

$$\mathrm{FBD}_{\mathcal{R}}(\mathcal{G}) = \|\mu_{\mathcal{G}} - \mu_{\mathcal{R}}\|_2^2 + \mathrm{Tr}\left(\Sigma_{\mathcal{G}} + \Sigma_{\mathcal{R}} - 2(\Sigma_{\mathcal{G}}\Sigma_{\mathcal{R}})^{1/2}\right), \tag{6}$$

where $\|\cdot\|_2$ denotes the Euclidean norm, $\mathrm{Tr}(\cdot)$ denotes the trace operator, and $(\Sigma_{\mathcal{G}}\Sigma_{\mathcal{R}})^{1/2}$ denotes the matrix square root of the product $\Sigma_{\mathcal{G}}\Sigma_{\mathcal{R}}$. In practice, we estimate $\mu_{\mathcal{G}}, \mu_{\mathcal{R}}, \Sigma_{\mathcal{G}}, \Sigma_{\mathcal{R}}$ from the data.

Maximum Mean Discrepancy (MMD) together with a positive-definite kernel κ, is used to compare the distributions of reference data embeddings and generated data embeddings. Assume that $\varphi_{\mathrm{E}}(\mathcal{G})$ and $\varphi_{\mathrm{E}}(\mathcal{R})$ are sampled from corresponding probability distributions $\lambda_{\mathcal{G}}$, $\lambda_{\mathcal{R}}$

$$\varphi_{\mathrm{E}}(\mathcal{G}) \sim \lambda_{\mathcal{G}},$$
$$\varphi_{\mathrm{E}}(\mathcal{R}) \sim \lambda_{\mathcal{R}}.$$

Then, the MMD between the generated data embeddings and the reference data embeddings is defined as

$$\mathrm{MMD}_{\kappa,\mathcal{R}}(\mathcal{G}) = \mathbb{E}_{X,X'\sim\lambda_{\mathcal{G}}}[\kappa(X,X')] + \mathbb{E}_{Y,Y'\sim\lambda_{\mathcal{R}}}[\kappa(Y,Y')] - 2\mathbb{E}_{X\sim\lambda_{\mathcal{G}},Y\sim\lambda_{\mathcal{R}}}[\kappa(X,Y)], \tag{7}$$

where $\kappa : \mathbb{R}^d \times \mathbb{R}^d \to \mathbb{R}$ is a positive-definite kernel function. The usual choice of κ is the Gaussian RBF kernel $\kappa(x,y) = \exp(-\|x-y\|_2^2/(2\sigma^2))$ (Jayasumana et al., 2024) or the rational quadratic kernel $\kappa(x,y) = \left(1 + \frac{\|x-y\|_2^2}{2\alpha}\right)^{-\alpha}$, where $\alpha > 0$ (Bińkowski et al., 2021).

In practice, we have access to i.i.d. samples from distributions $\lambda_{\mathcal{G}}$ and $\lambda_{\mathcal{R}}$

$$\{X_1, X_2, \ldots, X_n\} \overset{\text{i.i.d.}}{\sim} \lambda_{\mathcal{G}},$$
$$\{Y_1, Y_2, \ldots, Y_m\} \overset{\text{i.i.d.}}{\sim} \lambda_{\mathcal{R}},$$

and thus compute an unbiased estimator $\overline{\mathrm{MMD}}_{\kappa,\mathcal{R}}$ of $\mathrm{MMD}_{\kappa,\mathcal{R}}$

$$\overline{\mathrm{MMD}}_{\kappa,\mathcal{R}}(\mathcal{G}) = \frac{1}{n(n-1)}\sum_{i=1}^{n}\sum_{j\neq i}^{n}\kappa(X_i,X_j) + \frac{1}{m(m-1)}\sum_{i=1}^{m}\sum_{j\neq i}^{m}\kappa(Y_i,Y_j) - \frac{2}{nm}\sum_{i=1}^{n}\sum_{j=1}^{m}\kappa(X_i,Y_j). \tag{8}$$

Improved Precision and Recall uses intra-set k-th nearest neighbor distance to calibrate the binary classification of a point as a set member. We classify x as being included in set $\mathcal{R}$ if there exists $y \in \mathcal{R}$ such that x is closer to y than the k-th nearest neighbor of y from set $\mathcal{R}$. We follow Kynkäänniemi et al. (2019) and define the Improved Precision and Recall to measure similarity between sets $\varphi_{\mathrm{E}}(\mathcal{G})$ and $\varphi_{\mathrm{E}}(\mathcal{R})$ as

$$\mathrm{Precision}_{k,\mathcal{R}}(\mathcal{G}) = \frac{|\{x \in \mathcal{G} : \exists_{y\in\mathcal{R}} \|\varphi_{\mathrm{E}}(x) - \varphi_{\mathrm{E}}(y)\|_2 \le \|\varphi_{\mathrm{E}}(y) - \mathrm{NN}_{k,\varphi_{\mathrm{E}}}(y,\mathcal{R})\|_2\}|}{|\mathcal{G}|}, \tag{9}$$

$$\mathrm{Recall}_{k,\mathcal{R}}(\mathcal{G}) = \frac{|\{y \in \mathcal{R} : \exists_{x\in\mathcal{G}} \|\varphi_{\mathrm{E}}(y) - \varphi_{\mathrm{E}}(x)\|_2 \le \|\varphi_{\mathrm{E}}(x) - \mathrm{NN}_{k,\varphi_{\mathrm{E}}}(x,\mathcal{G})\|_2\}|}{|\mathcal{R}|}, \tag{10}$$

where $\mathrm{NN}_{k,\varphi_{\mathrm{E}}}(x,\mathcal{G})$ is an embedding of the k-th nearest neighbor of x from the set $\mathcal{G}$ under the embedding function φ_{E}.

Authenticity measures the fraction of generated samples that appear to be sampled from the approximated distribution, rather than being memorized and recalled training examples, i.e., near-copied (Alaa et al., 2022). Intuitively, generated samples that are closer to a training example than any other training data are considered memorized and therefore not authentic. Formally, for $x \in \mathcal{G}$ we define it's closest neighbor $y^*(x)$ from the reference set $\mathcal{R}$ as

$$y^*(x) = \mathrm{argmin}_{y\in\mathcal{R}} \|\varphi_{\mathrm{E}}(x) - \varphi_{\mathrm{E}}(y)\|_2,$$

and define the Authenticity metric as

$$\mathrm{Authenticity}_{\mathcal{R}}(\mathcal{G}) = \frac{|\{x \in \mathcal{G} : \|\varphi_{\mathrm{E}}(x) - \varphi_{\mathrm{E}}(y^*(x))\|_2 > \inf_{y\in\mathcal{R}\setminus\{y^*(x)\}}\{\|\varphi_{\mathrm{E}}(y) - \varphi_{\mathrm{E}}(y^*(x))\|_2\}\}|}{|\mathcal{G}|}. \tag{11}$$

Vendi Score is a reference-free diversity metric (Friedman and Dieng, 2023). It is defined as the exponential of the Shanon entropy of the trace-normalized similarity matrix's eigenvalues. The similarity matrix is induced by a positive semi-definite kernel function κ. Formally, for $\mathcal{G} = \{x_1, x_2, \ldots, x_n\}$ and $\mathbf{z} = (z_1, z_2, \ldots, z_n) = (\varphi_{\mathrm{E}}(x_1), \varphi_{\mathrm{E}}(x_2), \ldots, \varphi_{\mathrm{E}}(x_n))$, let $S_{ij}(\mathcal{G}) = \kappa(z_i, z_j)$ be the similarity matrix of set $\mathcal{G}$ with respect to some embedding model E and similarity kernel κ. Given the eigenvalues of the trace-normalized similarity matrix

$$\overline{\sigma}(S(\mathcal{G})) = \sigma\left(\frac{S(\mathcal{G})}{\mathrm{Tr}(\mathrm{S}(\mathcal{G}))}\right) = \{\lambda_1, \lambda_2, \ldots, \lambda_n\},$$

the Vendi Score is defined as

$$\mathrm{VENDI}_{\kappa}(\mathcal{G}) = \exp\left(-\sum_{i=1}^{n} \lambda_i \log \lambda_i\right), \tag{12}$$

where a higher score indicates more modes in the embedding space and therefore greater diversity. Note that $\sum_{\lambda\in\sigma(A)} \lambda = \mathrm{Tr}(A)$, hence the normalized eigenvalues $\overline{\sigma}(A)$ sum up to 1 and the entropy is well defined.

The usual choice of κ is the Gaussian kernel $\kappa(x, y) = \exp(-\|x - y\|_2^2/(2\sigma^2))$ for some $\sigma > 0$. We can approximate the spectrum of the similarity matrix $\sigma(S(\mathcal{G}))$ using Random Fourier Features of kernel κ. For a Gaussian kernel, fix a set of random feature vectors $\{\omega_1, \omega_2, \ldots, \omega_m\} \sim \mathcal{N}(0, \sigma^{-2} I_{d\times d})$ and define the Random Fourier Feature map $\phi : \mathbb{R}^d \to \mathbb{R}^{2m}$ as

$$\phi(z) = \sqrt{\frac{1}{m}} \left[\cos(\langle\omega_1, z\rangle), \sin(\langle\omega_1, z\rangle), \ldots, \cos(\langle\omega_m, z\rangle), \sin(\langle\omega_m, z\rangle)\right].$$

The resulting similarity matrix $\phi(\mathbf{z})\phi(\mathbf{z})^T = \langle\phi(z_i), \phi(z_j)\rangle_{ij} \in \mathbb{R}^{n\times n}$ has approximately the same spectrum as the original matrix $S_{ij}(\mathcal{G}) = \kappa(z_i, z_j)$ but conveniently shares the nonzero eigenvalues with the matrix $\phi(\mathbf{z})^T\phi(\mathbf{z}) \in \mathbb{R}^{2m\times 2m}$ which is beneficial considering computational savings when $2m < n$. This approximation was introduced in Ospanov et al. (2024). Denote the normalized eigenvalues of approximated similarity matrix $\{\tilde{\lambda}_1, \tilde{\lambda}_2, \ldots, \tilde{\lambda}_{2m}\} = \overline{\sigma}(\phi(\mathbf{z})^T\phi(\mathbf{z}))$ and define the Fourier Kernel Entropy Approximation (FKEA) of the Vendi Score as

$$\text{FKEA-VENDI}(\mathcal{G}) = \exp\left(-\sum_{i=1}^{n} \tilde{\lambda}_i \log \tilde{\lambda}_i\right). \tag{13}$$

Finally, one can generalize the above measure by using the more general Rényi entropy in the exponent

$$\text{FKEA-VENDI}_{\alpha}(\mathcal{G}) = \exp\left(\frac{1}{1-\alpha} \log\left(\sum_{i=1}^{n} \tilde{\lambda}_i^{\alpha}\right)\right). \tag{14}$$

Note that $\lim_{\alpha\to 1} \text{FKEA-VENDI}_{\alpha}(\mathcal{G}) = \text{FKEA-VENDI}(\mathcal{G})$.

1.2.3 Metrics from sequence properties

In this group, metrics use representation functions φ_{P} that assign some property to each element $x \in \mathcal{D}$. The metrics are computed using different aggregations of properties given by φ_{P}. We denote these metrics as property-based metrics.

Property moment is defined for scalar properties $\varphi_{\mathrm{P}} : \mathcal{D} \to \mathbb{R}$ and simply aggregates the property with mean and variance

$$\mathrm{ID}_{\varphi_{\mathrm{P}}}(\mathcal{G}) = \left[\frac{1}{|\mathcal{G}|}\sum_{x\in\mathcal{G}} \varphi_{\mathrm{P}}(x), \frac{1}{|\mathcal{G}|}\sum_{x\in\mathcal{G}} \left(\varphi_{\mathrm{P}}(x) - \frac{1}{|\mathcal{G}|}\sum_{x'\in\mathcal{G}} \varphi_{\mathrm{P}}(x')\right)^2\right]. \tag{15}$$

Hit-rate is defined for binary properties $\varphi_{\mathrm{P}} : \mathcal{D} \to \{0, 1\}$ and computes the fraction of positively-labeled elements

$$\text{Hit-rate}_{\varphi_{\mathrm{P}}}(\mathcal{G}) = \frac{|\{x \in \mathcal{G} : \varphi_{\mathrm{P}}(x) = 1\}|}{|\mathcal{G}|}. \tag{16}$$

Threshold which is a special case of Hit-rate, is defined for scalar properties $\varphi_{\mathrm{P}} : \mathcal{D} \to \mathbb{R}$ and computes the fraction of generated elements whose property exceeds a given threshold

$$\mathrm{Threshold}_{\varphi_{\mathrm{P}},c}(\mathcal{G}) = \frac{|\{x \in \mathcal{G} : \varphi_{\mathrm{P}}(x) > c\}|}{|\mathcal{G}|}. \tag{17}$$

Hypervolume is used in multi-objective optimization, when several and often conflicting properties are optimized (Zitzler and Thiele, 1999). The Hypervolume Indicator (HVI) is defined for positive vector-valued properties $\varphi_{\mathrm{P}} : \mathcal{D} \to \mathbb{R}_+^k$ and measures the volume under the Pareto front of the set $\varphi_{\mathrm{P}}(\mathcal{G})$

$$\mathrm{HVI}_{\varphi_{\mathrm{P}}}(\mathcal{G}) = \mathrm{vol}_{\mathbb{R}_+^k}\left(\mathrm{Pareto}(\varphi_{\mathrm{P}}(\mathcal{G}))\right) = \mathrm{vol}_{\mathbb{R}_+^k}\left(\bigcup_{x\in\mathcal{G}}\{y \in \mathbb{R}_+^k : \forall_{i\in\{1,2,\ldots,n\}} y_i \le \varphi_{\mathrm{P}}(x)_i\}\right). \tag{18}$$

Alternatively, one can measure the volume of the convex hull

$$\mathrm{Convex}(\varphi_{\mathrm{P}}(\mathcal{G})) = \left\{\sum_{g\in\mathcal{G}} \lambda_g g : \lambda_g \ge 0, \sum_{g\in\mathcal{G}} \lambda_g = 1\right\}.$$

Conformity score can be defined for any property function $\varphi_{\mathrm{P}} : \mathcal{D} \to Y$. It uses a conformity measure A which jointly assigns the conformity scores a for each pair $(x, \varphi_{\mathrm{P}}(x))$ given the whole set $\mathcal{G}$. We only require A to be permutation equivariant. Formally, for $\mathcal{G} = \{x_1, x_2, \ldots, x_n\}$ and $\mathcal{R} = \{y_1, y_2, \ldots, y_m\}$, let

$$A\left((x_i, \varphi_{\mathrm{P}}(x_i))_{i=1}^n\right) = (a_i)_{i=1}^n,$$
$$A\left((y_i, \varphi_{\mathrm{P}}(y_i))_{i=1}^m\right) = (b_i)_{i=1}^m,$$

denote the conformity scores of the generated set and reference set, respectively. Since A is permutation equivariant, each x_i has a unique score assigned $a_i = a(x_i)$ which depends only on the value of x and not the index i. Similarly $b_i = b(y_i)$.

The Conformity Score metric is defined as the fraction of generated-reference pairs for which the conformity score of the generated sample is greater than the conformity score of the reference sample

$$\mathrm{CS}_{A,\mathcal{R}}(\mathcal{G}) = \frac{|\{(x, y) \in \mathcal{G} \times \mathcal{R} : a(x) \ge b(y)\}|}{|\mathcal{G}||\mathcal{R}|}. \tag{19}$$

In practice, A is the log-likelihood of the joint density over a user-specified set of sequence properties, and the Conformity Score is averaged over K-folds sampled from the reference set (Frey et al., 2024).

KL-divergence between the property distributions of the reference set and the generated set can be computed for $\varphi_{\mathrm{P}} : \mathcal{D} \to Y$ where Y is either a discrete space or a vector space $\mathbb{R}^k$. Using a kernel density estimator of choice, we approximate the distributions $\lambda_{\mathcal{G}}$ and $\lambda_{\mathcal{R}}$

$$\varphi_{\mathrm{P}}(\mathcal{G}) \sim \lambda_{\mathcal{G}} = p_{\mathcal{G}}\lambda,$$
$$\varphi_{\mathrm{P}}(\mathcal{R}) \sim \lambda_{\mathcal{R}} = p_{\mathcal{R}}\lambda,$$

which are absolutely continuous with respect to some common measure λ. Then we use the usual definition of Kullback–Leibler divergence

$$\mathrm{KL}_{\mathcal{R}}(\mathcal{G}) = D_{\mathrm{KL}}(\lambda_{\mathcal{G}}||\lambda_{\mathcal{R}}) = \int_Y p_{\mathcal{G}} \log \frac{p_{\mathcal{G}}}{p_{\mathcal{R}}} d\lambda. \tag{20}$$

In practice, the KL-divergence is approximated using Monte-Carlo sampling.

1.3 Limitations of the metrics

Metric failure modes While embedding-based metrics generalize across multiple biological domains, they rely on two key assumptions that, when violated, can cause metric deterioration. The first is a structural assumption about the embedding space, e.g., FBD assumes that embedding point clouds follow a Gaussian distribution, and MMD assumes a Gaussian kernel. The second is a sensitivity to the choice of embedding model itself. If the embedding space is not ordered as the metric expects, results can become unreliable. More broadly, Räisä et al. (2025) demonstrate that all current fidelity and diversity metrics fail at one or more sanity checks, exhibiting issues such as lack of outlier robustness, mode collapse, and poor behavior in high-dimensional embedding spaces. Improved precision and improved recall are, e.g., not robust towards outliers. Thus, designing more robust metrics remains an important research direction.

Hyperparameter sensitivity Beyond distributional assumptions, several metrics require hyperparameters whose optimal values are not universal. MMD requires bandwidth selection, and improved precision/recall requires specifying k in k-NN. Strashnov et al. (2024) find that a bandwidth of 10 is a reasonable fit for MMD on protein sequences, but observe that optimal hyperparameter values are sensitive to the embedding model and do not transfer reliably across all settings. We therefore recommend performing an initial hyperparameter sensitivity analysis for the biological domain of interest.

Sample size sensitivity An added complexity of applying metrics is their sensitivity to sample size. Strashnov et al. (2024) evaluates the robustness of several metrics on protein sequences, including MMD, FBD, improved precision and improved recall, and finds that the metrics depend on sample size. Thus, to ensure sample size biased is eliminated, all models should evaluate the same number of sequences. Additionally, Strashnov et al. (2024) recommend to use at least 1,024 samples as it provides stable approximations for protein sequences.

Computational cost Finally, a practical consideration when selecting metrics is computational cost. Metrics that require structure prediction, such as pLDDT and self-consistency perplexity, are substantially more expensive than sequence-based metrics, as they require running a folding model for every generated sequence. We therefore recommend reserving expensive structure-based metrics for final evaluations where computational resources permit.

1.4 Diagnostics

Embedding-based metrics require an embedding model to map sequences to fixed-size vector representations. Since a misaligned embedding model will likely produce unreliable or uninterpretable metrics, we include two diagnostics to assess how well an embedding model's sequence representations align with the sequence features of interest.

1.4.1 k-NN feature-alignment score

The k-nearest neighbor (k-NN) feature-alignment score (Rissom et al., 2025) evaluates an embedding model's alignment with labels assigned by a discrete valued function $y : \mathcal{R} \to \{1, 2, \dots, K\}$. Let $\mathrm{E} : \mathcal{D} \to \mathbb{R}^d$ denote the embedding model mapping a sequence to a fixed-size vector representation and $\mathrm{NN}_{k,E}(x)$ denote the set of k nearest neighbors under embedding E. The k-NN feature-alignment score of the embedding model E is defined as

$$\mathrm{FAS}_{k,\mathcal{R}}(\mathrm{E}) = \frac{1}{|\mathcal{R}|} \sum_{x \in \mathcal{R}} \frac{|\{x' \in \mathrm{NN}_{k,E}(x) : y(x') = y(x)\}|}{k}. \tag{21}$$

The k-NN feature-alignment score yields a value between 0 and 1. An embedding model aligning well with the sequence feature(s) of interest has a value close to 1.

1.4.2 Spearman alignment score

The Spearman alignment score (Rissom et al., 2025) evaluates an embedding model's alignment with sequence properties when the properties are continuous. The Spearman alignment score is defined as the Spearman correlation between the pairwise distance between sequences in the embedding space and the feature space, respectively.

1.5 Case studies

1.5.1 Case study 1: Benchmarking antimicrobial peptide design models

In this case study, we use `seqme` to benchmark five existing generative models for antimicrobial peptide (AMP) design, obtaining 10,000 generated sequences from each. The models span three generative paradigms: diffusion models, generative adversarial networks, and conditional variational autoencoders.

AMP-Diffusion (Torres et al., 2025) is a latent diffusion model that denoises Gaussian noise into ESM2 embeddings subsequently decoded into amino acid sequences. It employs a Markov chain-based forward process that progressively corrupts ESM2 peptide embeddings with Gaussian noise, and trains a neural network to reverse this process, reconstructing clean embeddings from noised inputs. The frozen ESM2 language model head then decodes the recovered embeddings into amino acid sequences, enriching the diffusion process with prior evolutionary knowledge encoded in the pretrained protein language model.

MMCD (Wang et al., 2024) is a multi-modal diffusion model that co-generates sequences alongside backbone coordinates. It constructs separate sequence-modal and structure-modal diffusion models and couples them through a contrastive learning objective applied at each timestep, where an inter-contrastive term maximises the agreement between sequence and structure embeddings of the same peptide, and an intra-contrastive term maximises the disagreement between embeddings of therapeutic and non-therapeutic peptides.

AMP-GAN v2 (Van Oort et al., 2021) is a bidirectional conditional generative adversarial network in which the generator maps samples from a latent Gaussian distribution to AMP sequences, the discriminator distinguishes real from generated sequences conditioned on binary metadata vectors encoding properties such as microbial target and mechanism of action, and a third encoder network maps real sequences back into the generator's latent space to enable iterative latent space manipulation.

PepCVAE (Das et al., 2018) is a conditional variational autoencoder that adopts a semi-supervised training strategy, in which a GRU-based encoder–decoder learns a continuous latent space from a large corpus of unlabeled peptides, while a jointly trained LSTM-based AMP classifier enforces a disentangled antimicrobial attribute space, allowing controllable generation by conditioning the decoder on the desired activity label.

HydrAMP (Szymczak et al., 2023) is a conditional variational autoencoder that augments the standard cVAE objective with a Jacobian disentanglement regularizer and latent reconstruction loss, and introduces two auxiliary classifiers predicting antimicrobial activity and minimum inhibitory concentration whose gradients guide the decoder to separate the antimicrobial condition from the sequence latent vector. A temperature parameter further controls the trade-off between generation diversity and proximity to a prototype sequence, supporting both unconstrained and analogue generation modes.

In addition to the models, we have acquired sequences from two databases. DBAASP (Pirtskhalava et al., 2020) is a curated database of experimental validated antimicrobial peptides. UniProt (Consortium, 2024) is a large-scale general protein and peptide database that captures the diversity of naturally occurring sequences. Both reference datasets were filtered to sequences of 8–50 amino acids consistent with typical AMP length, yielding 13,926 sequences from DBAASP and 2,933,310 sequences from UniProt. Lastly, we randomly sample 10,000 sequences from each of the databases.

Metric selection. We now provide a discussion of the metric selection strategy. We first note that both sequence-based and embedding-based metrics provide general quality insights that are always useful to compute to evaluate the design based on different aspects. However, the actual choice of the cut-off thresholds for the metrics, the choice of the embedding model for the embedding-based metrics and finally, the choice of the property-based metrics depend on the user-defined task and are left for the user to define. To guide the user choices, here, we first discuss the general phenomenon of realism-idealism tradeoff in sequence design, which is important to be aware of. Next, for the embedding-based metrics, we illustrate the analysis assessing the suitability of candidate embedding models, then examine metric correlation and redundancy. Finally, both for embedding-based and property-based metrics, we determine their correlation with biologically meaningful properties.

The realism-idealism tradeoff. A central challenge in evaluating *de novo* sequence generation is the tension between *realism* and *idealism*: sequences optimized purely for a single surrogate property, such as predicted potency against bacteria, risk reward hacking, whereby high scores on that surrogate are achieved at the expense of broader viability—for example by producing sequences that cannot fold correctly and would therefore be non-functional in practice. In this benchmark, we therefore want to include metrics which cover both realism and idealism. To be concrete, we are interested in scoring the generative models based on whether they generate plausible, well-formed peptides with antimicrobial properties. Additionally, we want to reward sequence diversity, as models outputs should span a broad region of sequence space rather than collapsing to a narrow set of near-duplicate solutions. Based on this design preference, we now analyze the embedding models and metrics best aligning with these goals.

Choice of embedding model. For this benchmark, a well-aligned embedding model should be able to distinguish between AMPs and non-AMPs. We treat DBAASP sequences as AMPs and UniProt sequences as non-AMPs, and evaluate the embedding models by computing the k-NN feature-alignment score across several neighborhood sizes, which uncovers the separation at a local and global resolution. Furthermore, we visualize the embeddings projected onto the first two principle components. We compare ESM2 (150M parameter checkpoint), Hyformer (35M parameter peptide checkpoint) and embeddings derived from physico-chemical properties, namely by computing the charge, hydrophobicity and mass for each sequence. As shown in Fig. S1, Hyformer provides the best separation both locally and globally between AMPs and non-AMPs. ESM2 provides a strong local separation but poor global separation. Lastly, the embeddings based on physico-chemical properties performs poorly at local separation but does not deteriorate

further at global separation. The PCA projections in Fig. S2 corroborate these findings on global separation. Based on these findings, we choose to use Hyformer embeddings for the remainder of the analysis and benchmark.

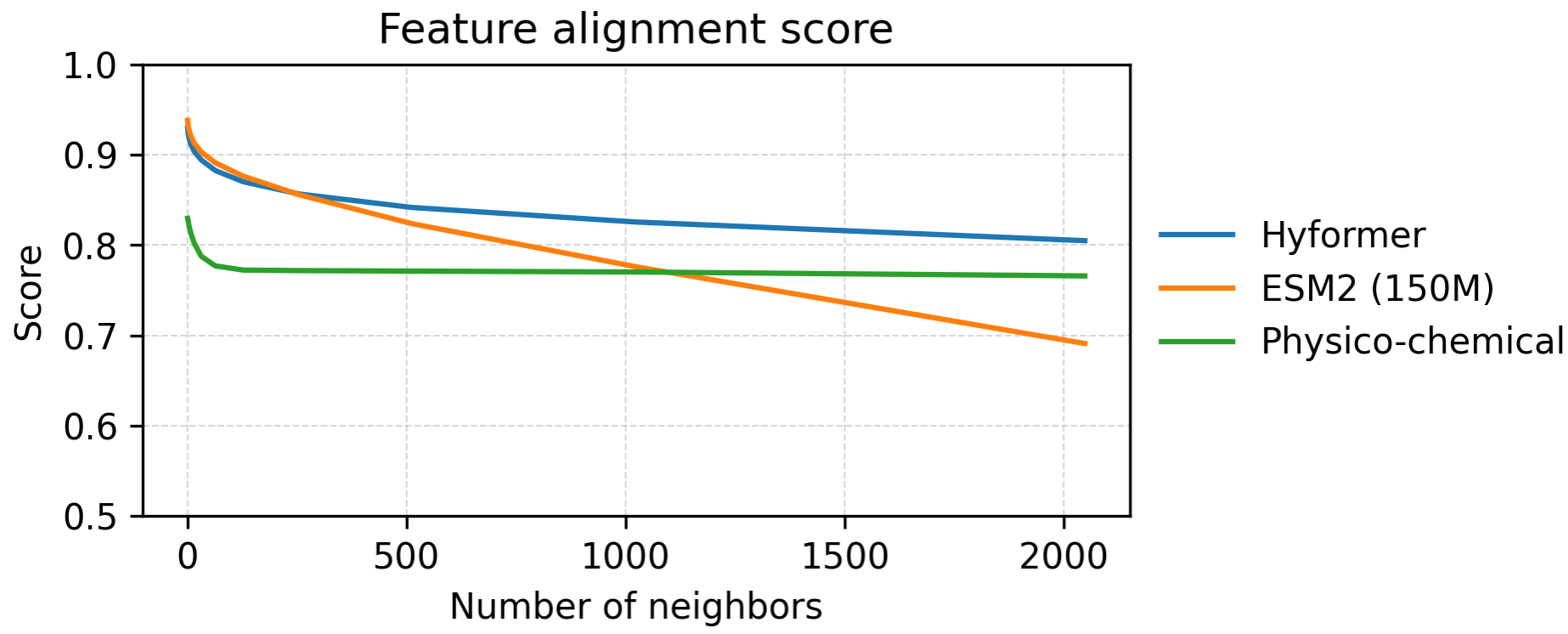

Figure S1: Feature-alignment score using k-NN ($k = 1$–2048) across embedding models, measuring separation of DBAASP and UniProt sequences. Score = fraction of correctly assigned sequences based on majority vote of nearest neighbors. Higher values indicate better separation between the groups.

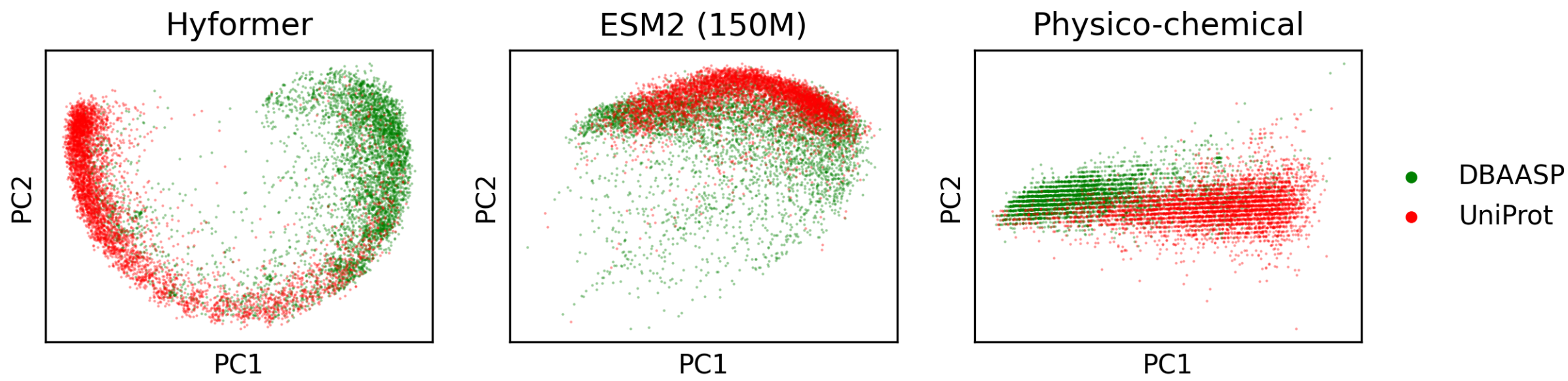

Figure S2: PC embeddings for different embedding models. Each point represents the embedding of a sequence, with colors indicating the corresponding data source.

Embedding metric correlation and redundancy. To examine metric correlation and redundancy, we plot a cross-correlation matrix between metrics using 5,000 sequences from each of the data sources: DBAASP, UniProt, the 5 generative models, and also DBAASP and UniProt where the order of amino acids has been randomly permuted for each sequence. We compare the correlation between the following metrics: Diversity, FKEA, Precision (DBAASP) using 12-nearest neighbors, Recall (UniProt) using 12-nearest neighbors, MMD (UniProt), MMD (DBAASP), FBD (UniProt), and FBD (DBAASP). The reference sequences in UniProt and DBAASP do not overlap with the sequences used for evaluation. Additionally, we compute the Spearman correlation.

According to Fig. S3, the MMD and FBD are significantly correlated ($p\text{-value} < 0.001$) when using the same reference dataset (UniProt or DBAASP) and embedding model (Hyformer). Additionally, Recall (UniProt) and MMD (UniProt), Recall (UniProt) and FBD (UniProt), FKEA and MMD (DBAASP), and FKEA and FBD (DBAASP) are also statistically significantly correlated ($p\text{-value} < 0.05$). None of the remaining metrics are statistically significantly correlated ($p\text{-value} < 0.05$).

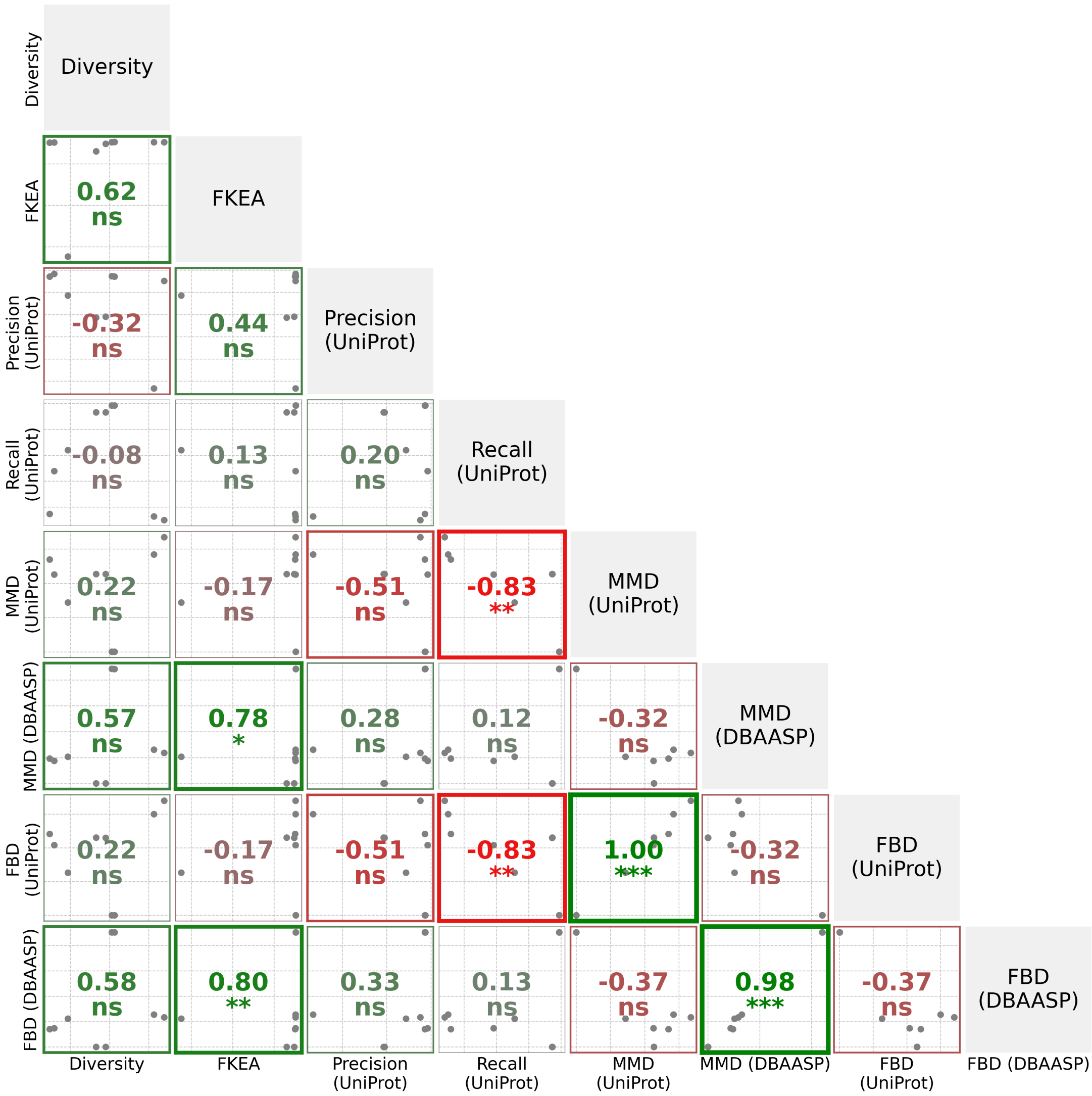

Figure S3: Cross-correlation matrix for different metrics in the AMP benchmark. Each point corresponds to sequences from a data source.

Correlation with meaningful biological features. We finish this analysis by verifying that MMD and FBD correlate with biological meaningful features. We follow a setup similar to Preuer et al. (2018), which analyzes the Frechet ChemNet Distance for small molecules. We use Hyformer (35M peptide checkpoint) as embedding model. We use three reference datasets; DBAASP, the AMP dataset from Veltri et al. (2018), and the non-AMP dataset from Veltri et al. (2018). The two latter datasets are also referred to as the Veltri dataset. We use sequences from DBAASP as reference sequences, and compute the scores on the AMP and non-AMP dataset from Veltri et al. (2018). This yields a fair comparison as the sequences from Veltri et al. (2018) constituted a separate dataset from DBAASP. We sample 1,700 from each of the datasets for the evaluation. We compare the MMD and FBD with AMPlify and amPEPpy predictions, KL-divergence based on charge and KL-divergence using hydrophobocity. For the AMP classifiers, AMPlify and amPEPpy, we label a sequence as AMP if the output prediction is above 0.75 otherwise as a non-AMP.

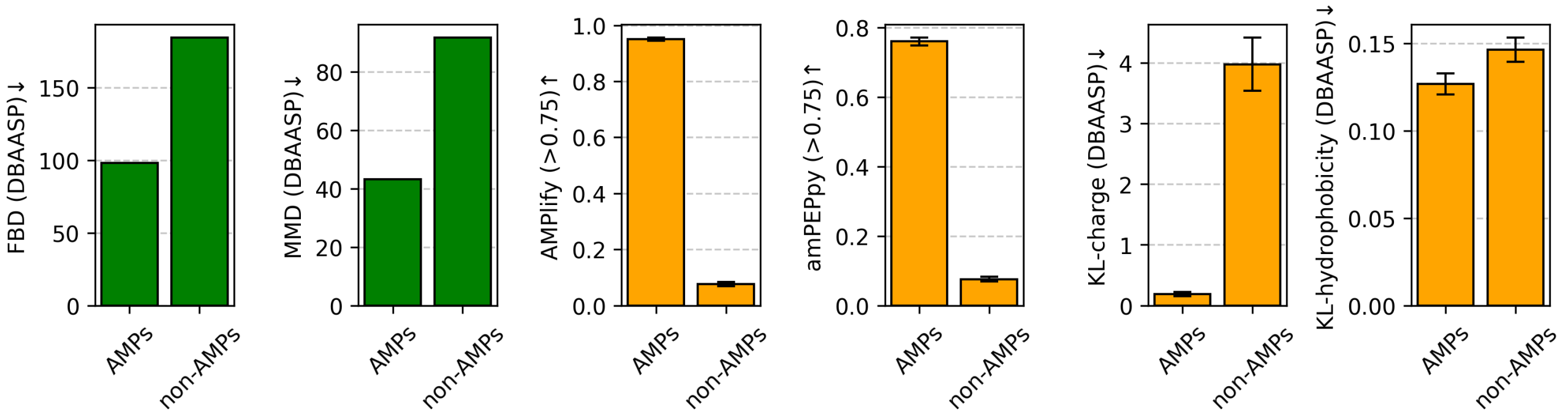

Figure S4: Cross-correlation matrix for different metrics in the AMP benchmark. Each point corresponds to sequences from a single model.

Fig. S4 shows that a low FBD or MMD yields a high probability of the sequences being AMP and the sequences has similar physico-chemical properties as the reference sequences, namely the AMPs from DBAASP. Thus, MMD and FBD can capture meaningful biological features, when using an appropriate embedding model and reference dataset.

Benchmark. Based on the preceding analysis, we evaluate all 5 models using the following metrics: Diversity, FKEA, FBD (DBAASP), FBD (UniProt), and the median minimum inhibitory concentration (MIC) predicted by AMPredictor. *Diversity* is computed via a k-nearest-neighbour approximation with $k = 200$. The embedding-based metrics *FKEA* and *FBD* are derived from representations produced by the 35M-parameter Hyformer model (Izdebski et al., 2025), while AMPredictor (Dong et al., 2025) estimates the MIC of each sequence against a reference bacterial strain.

For each model, the 10,000 generated sequences are partitioned into five non-overlapping splits of 2,000 sequences; per-metric means and standard deviations are then aggregated across splits using `seqme`'s *Fold* utility—a standard approach for quantifying evaluation uncertainty in generative models (Rodriguez et al., 2025; Kmicikiewicz et al., 2025; Tang et al., 2025). AMPredictor-based MIC estimates are computed on the full set of 10,000 sequences without splitting, which, where applicable, enables pairwise statistical comparisons between models.

Finally, we perform non-dominated (Pareto) ranking computed using `seqme` jointly over *FBD* (DBAASP) and AMPredictor-predicted MIC values; ranks are assigned iteratively by identifying the non-dominated set, removing it, and repeating until all methods have been ranked.

Method	Diversity (↑)	FKEA (↑)	FBD (DBAASP) (↓)	FBD (UniProt) (↓)	AMPredictor (↓)	Rank (↓)
AMP-Diffusion	0.82 ± 0.01	1259 ± 17	110 ± 5	$\mathbf{251 \pm 9}$	$\underline{12.4}$	**1**
MMCD	0.81 ± 0.01	$\mathbf{1344 \pm 1}$	$\underline{75 \pm 2}$	$\underline{410 \pm 6}$	27.8	2
AMP-GAN	0.81 ± 0.01	$\mathbf{1344 \pm 1}$	$\mathbf{70 \pm 3}$	477 ± 7	14.6	**1**
HydrAMP	$\underline{0.86 \pm 0.01}$	$\mathbf{1344 \pm 1}$	129 ± 3	595 ± 2	**11.8**	**1**
PepCVAE	$\mathbf{0.87 \pm 0.01}$	$\mathbf{1344 \pm 1}$	119 ± 2	674 ± 2	22.5	2

Table S1: Mean and std. across 5 runs, each run with 2000 sequences, except for AMPredictor where all 10,000 sequences are evaluated in a single run. Best values are shown in **bold**, and second best values are underlined. Table is generated using `seqme`.

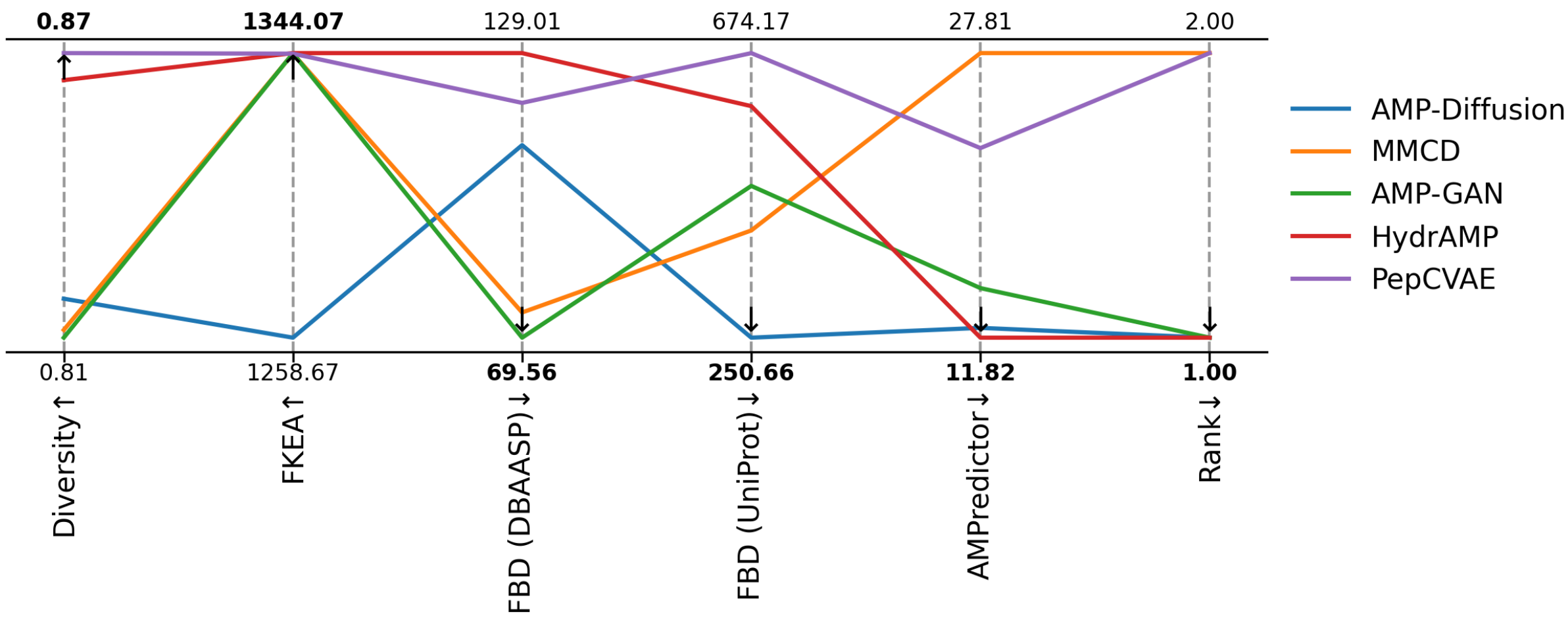

Figure S5: Benchmark results visualized using `seqme`'s parallel coordinates plot function. Each metric is a coordinate and lines represent individual model performance. Arrows indicate whether a metric ought to be maximized or minimized.

Table S1 and Fig. S5 summarize the benchmark results. The VAE-based models, HydrAMP and PepCVAE, generate the most diverse sequences, yet exhibit poor distributional fidelity as measured by FBD against both UniProt and DBAASP. With the exception of AMP-Diffusion, all models achieve identical embedding-level diversity as measured by FKEA. AMP-GAN, by contrast, achieves the best FBD against DBAASP and attains Pareto rank 1 jointly with HydrAMP and AMP-diffusion. To test whether the difference in median predicted MIC between the two models yielding the lowest MIC, namely HydrAMP and AMP-Diffusion, is statistically significant, we perform a Mann-Whitney U test. We find that HydrAMP generates sequences with significantly lower predicted MIC than AMP-Diffusion (p-value < 0.001).

Taken together, no single model dominates across all metrics, underscoring the value of a comprehensive multi-metric evaluation. Nevertheless, HydrAMP presents the most favorable balance of predicted potency and diversity, making it the recommended choice when antimicrobial activity is the primary design objective, while AMP-GAN is preferable when distributional alignment with experimentally validated AMPs is prioritized.

1.5.2 Case study 2: Acquiring high-quality mRNA data

The second case study illustrates how `seqme` can be applied to monitor the process of selecting a high quality subset of mRNA sequences from a larger pool. This case is motivated by a typical scesnario, where a small subset of computationally designed sequences needs to be selected for wet-lab validation given a fixed experimental budget.

To select the bag of sequences, we run a genetic algorithm (see Algorithm 1). Starting from an initial pool of mRNA sequences, the genetic algorithm iteratively evaluates and ranks bags of sequences using metrics from `seqme`. A bag's fitness is defined as the sequences' diversity in the embedding space measured with *FKEA* and average stability. We use RNA-FM (Chen et al., 2022) as the embedding model for *FKEA*, and the minimum free energy of the predicted secondary structure as a measure of sequence instability (Zhang et al., 2023), computed using RNAfold from the ViennaRNA package (Lorenz et al., 2011). This fitness function encourages the selection of bags that are both diverse and structurally stable. The output of the genetic algorithm is the set of non-dominated bags, and it is the user's task to select the final bag of sequences from these non-dominated bags. The genetic algorithm is presented in Algorithm 1. The lines highlighted in green indicate the use of functionality provided by `seqme`.

Algorithm 1: GABS: Genetic Algorithm for Bag Selection

Input: Sequence pool, M metrics, bag size J, history size K, number of iterations N.
Output: Non-dominated bags of biological sequences.

Sample without replacement a bag with J sequences from the pool.
for $i = 1$ **to** N **do**
  Evaluate the bag using the M metrics.
  Extract the non-dominated set of bags from the previous K iterations.
  Randomly sample a bag from the non-dominated set.
  Form the next bag by sampling $(1 - F) \times J$ sequences from the bag and $F \times J$ sequences from the pool.
Extract the non-dominated bags.
return Non-dominated bags of sequences.

We run Algorithm 1 with the following parameters: number of iterations $N = 50{,}000$, bag size $J = 1{,}000$, history size $K = 500$, and the pool fraction F defined by a bounded decay function $F = f(i)$, where $f(t) = N_1 + (N_0 - N_1)\exp(-rt)$ with initial value $N_0 = 0.05$, final value $N_1 = 1/J = 0.001$, and decay rate $r = 0.0005$. The sequence pool contains 12,000 randomly sampled mRNA sequences from the data used in Xiong et al. (2025), which was used for pre-training their mRNA model. The total run-time is 4 hours. Results of running the algorithm is depicted in Figure S6.

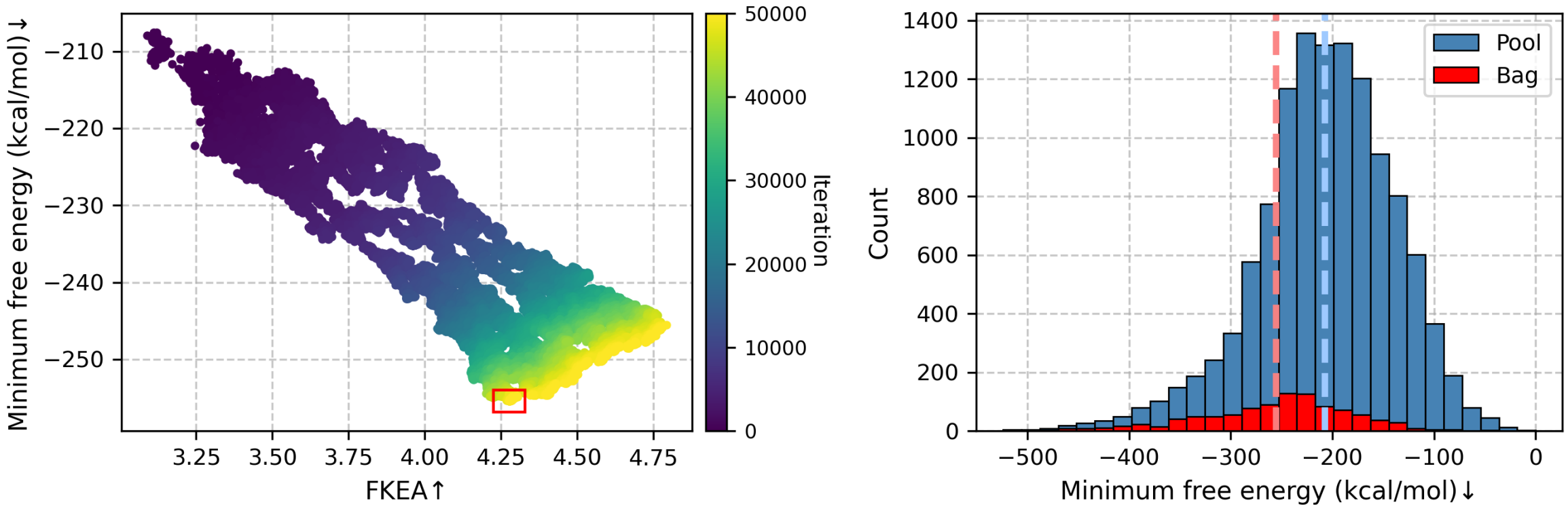

Figure S6: **Left**: Fitness of the bags. Each point is a bag. The bag in the red box is the bag in the right figure. **Right**: Minimum free energy distribution of the bag with lowest average minimum free energy and the pool. The average minimum free energy for each group is visualized with a vertical dashed line.

According to Figure S6, left, bag fitness improves over the iterations of running the genetic algorithm and produces a non-dominated set with a trade-off between average minimum free energy and diversity as measured by *FKEA*. By sampling the bag with lowest average minimum free energy, we observe that its minimum free-energy distribution is left-shifted relative to that of the full pool (Figure S6, right). Hence, the mRNA sequences selected by the algorithm are on average more stable than those in the pool. To summarize, we demonstrate the capabilities of `seqme` for iterative sequence selection for multi-objective optimization through its utilties. Importantly, this approach is not limited to mRNA and transfers readily to other biological sequence types and fitness functions, underscoring the generality of `seqme` for this kind of task.

1.6 Comparison to existing frameworks

While existing benchmarks for biological sequence design exist for specific applications, a general framework to easily create new benchmarks is lagging. Existing tools are purpose-built for a fixed set of tasks and metrics, leaving researchers without a reusable backbone when moving to new sequence types or design objectives. `seqme` addresses this gap by providing a modular, extensible toolbox that combines common evaluation metrics, embedding models for all major biological domains, and a flexible interface for adding new metrics and models — enabling new benchmarks to be readily constructed on top of it.

For **small molecules**, GuacaMol (Brown et al., 2019) and MOSES (Polykovskiy et al., 2020) are established benchmarking platforms for de novo molecular generation, evaluating models on metrics such as validity, novelty, uniqueness, and Fréchet ChemNet Distance. For **proteins**, PDBench (Castorina et al., 2023) evaluates fixed-backbone sequence design methods beyond simple sequence recovery by validating designs secondary structure. While these frameworks have driven progress within their respective domains, they are purpose-built tools rather than a convenient

evaluation toolbox: Each is restricted to a single molecular class, hardcodes a fixed metric set, and provides no mechanism to extend evaluation to new sequence types or incorporate new scoring models. Consequently, when a researcher moves to a new domain or design task, the work of assembling a benchmark must begin from scratch. Notably, this is precisely the situation for **mRNA** and regulatory **DNA** design, where no shared benchmark framework exists and recent generative methods each resort to bespoke evaluation protocols using metrics such as codon adaptation index, minimum free energy, and oracle-based activity scores (Zhang et al., 2023; Gong et al., 2023).

`seqme` is designed from the ground up as a reusable backbone for benchmark construction. Rather than providing a single fixed benchmark, it supplies the building blocks—sequence-based, embedding-based, and property-based metrics, together with domain-specific embedding models for small molecules, DNA, RNA, peptides, and proteins—from which benchmarks for new tasks and modalities can be assembled with minimal effort. This distinguishes `seqme` from GuacaMol, MOSES, and PDBench, which offer a benchmark but not a framework, and directly addresses the absence of any benchmarking infrastructure for emerging design domains such as mRNA and regulatory DNA.

1.7 Dependencies and licence

`seqme` has the following dependencies: moocore (LGPL-2.1), modlAMP (BSD 3-Clause) (Müller et al., 2017), scikit-learn (BSD 3-Clause) (Pedregosa et al., 2011), UMAP-learn (BSD 3-Clause) (McInnes et al., 2018), Transformers (Apache-2.0) (Wolf et al., 2020), scipy (BSD 3-Clause) (Virtanen et al., 2020), PyTorch2 (custom) (Ansel et al., 2024), polyleven (MIT), matplotlib (custom) (Hunter, 2007), numpy (custom) (Harris et al., 2020), pandas (BSD 3-Clause) (The pandas development team), tqdm (MIT).

All dependencies are compatible with `seqme`'s BSD 3-clause license. To satisfy a BSD 3-clause license, models with incompatible licenses are not directly included in `seqme` but are accessible through third-party repositories and act as optional plugins. Likewise, models with dependency conflicts are not directly included in `seqme` but made compatible with `seqme`'s third-party interface, which supports separate virtual Python environments.

2 Supplementary Tables

Table S2: Supported metrics by sequence type

**Metrics**	**Protein**	**Peptide**	**RNA**	**DNA**	**Small Molecule**
Diversity	✓	✓	✓	✓	×
Uniqueness	✓	✓	✓	✓	✓
Novelty	✓	✓	✓	✓	✓
N-Gram Jaccard Similarity	✓	✓	✓	✓	×
Fréchet Biological Distance	✓	✓	✓	✓	✓
Maximum Mean Discrepancy	✓	✓	✓	✓	✓
Improved Precision	✓	✓	✓	✓	✓
Improved Recall	✓	✓	✓	✓	✓
Authenticity	✓	✓	✓	✓	✓
FKEA	✓	✓	✓	✓	✓
Property Moment	✓	✓	✓	✓	✓
Hit-rate	✓	✓	✓	✓	✓
Hypervolume	✓	✓	✓	✓	✓
Conformity score	✓	✓	✓	✓	✓
KL-divergence	✓	✓	✓	✓	✓

Table S3: Models currently included in `seqme`. * = models included in third-party repositories and ported to adhere to `seqme`'s interface. For an up-to-date list, check https://seqme.readthedocs.io/en/stable/api/models_index.html.

	Model	Description
**Embedding**	ESM-2 (Lin et al., 2022)	Masked language model with protein and peptide checkpoints.
	RNA-FM (Chen et al., 2022)	RNA language model trained on mRNA and ncRNA sequences.
	GENA-LM (Fishman et al., 2025)	A family of language models for long DNA sequences trained on human DNA sequence.
	Hyformer (Izdebski et al., 2025)*	Hybrid language model with small molecule, peptide and antimicrobial peptide checkpoint.
	k-mer frequency	Frequency of pre-specified k-mers of a sequence.
**Property**	Physico-chemical properties	10 physico-chemical properties of peptides/proteins and 4 descriptors for small molecules.
	AMPredictor (Dong et al., 2025)*	A regresssion model predicting minimum inhibitory concentration (MIC) against bacteria.
	AMPlify (Li et al., 2022)*	Ensemble model predicting the probability a sequence has antimicrobial properties.
	amPEPpy (Lawrence et al., 2020)*	Random forest model predicting the probability a sequence has antimicrobial properties.
	ESM-2 (Lin et al., 2022)	Masked language model for proteins (and proteins) computing a sequences pseudo-perplexity.
	ESMFold (Lin et al., 2022)*	Protein language model predicting proteins atom-wise coordinates, i.e., 3D structure, predicted local distance difference test (pLDDT), predicted alignment error (pAE) and predicted Template Modeling score (pTM).
	ESM-IF1(Hsu et al., 2022)*	A protein inverse folding model that enables computation of self-consistency perplexity (scPerplexity) and sequence recovery when combined with a protein folding model, e.g., ESMFold.
	GENA-LM (Fishman et al., 2025)	Family of DNA language models finetuned to predict splice sites and promoter regions.
	Hyformer (Izdebski et al., 2025)*	Hybrid language model predicting sequence perplexity and probability a sequence has antimicrobial properties.

References

A. M. Alaa, et al. How faithful is your synthetic data? sample-level metrics for evaluating and auditing generative models, 2022. URL https://arxiv.org/abs/2102.08921.

J. Ansel, et al. PyTorch 2: Faster Machine Learning Through Dynamic Python Bytecode Transformation and Graph Compilation. In *29th ACM International Conference on Architectural Support for Programming Languages and Operating Systems, Volume 2 (ASPLOS '24)*. ACM, Apr. 2024. doi: 10.1145/3620665.3640366. URL https://docs.pytorch.org/assets/pytorch2-2.pdf.

M. Bińkowski, et al. Demystifying mmd gans, 2021. URL https://arxiv.org/abs/1801.01401.

N. Brown, et al. Guacamol: Benchmarking models for de novo molecular design. *Journal of Chemical Information and Modeling*, 59(3):1096–1108, Mar. 2019. ISSN 1549-960X. doi: 10.1021/acs.jcim.8b00839. URL http://dx.doi.org/10.1021/acs.jcim.8b00839.

L. V. Castorina, et al. Pdbench: evaluating computational methods for protein-sequence design. *Bioinformatics*, 39 (1):btad027, 01 2023. ISSN 1367-4811. doi: 10.1093/bioinformatics/btad027. URL https://doi.org/10.1093/bioinformatics/btad027.

J. Chen, et al. Interpretable rna foundation model from unannotated data for highly accurate rna structure and function predictions, 2022. URL https://arxiv.org/abs/2204.00300.

T. U. Consortium. Uniprot: the universal protein knowledgebase in 2025. *Nucleic Acids Research*, 53(D1):D609–D617, 11 2024. ISSN 1362-4962. doi: 10.1093/nar/gkae1010. URL https://doi.org/10.1093/nar/gkae1010.

P. Das, et al. Pepcvae: Semi-supervised targeted design of antimicrobial peptide sequences, 2018. URL https://arxiv.org/abs/1810.07743.

R. Dong, et al. Exploring the repository of de novo-designed bifunctional antimicrobial peptides through deep learning. *eLife*, 13:RP97330, mar 2025. ISSN 2050-084X. doi: 10.7554/eLife.97330. URL https://doi.org/10.7554/eLife.97330.

V. Fishman, et al. Gena-lm: a family of open-source foundational dna language models for long sequences. *Nucleic Acids Research*, 53(2):gkae1310, 01 2025. ISSN 1362-4962. doi: 10.1093/nar/gkae1310. URL https://doi.org/10.1093/nar/gkae1310.

N. C. Frey, et al. Protein discovery with discrete walk-jump sampling, 2024. URL https://arxiv.org/abs/2306.12360.

D. Friedman, et al. The vendi score: A diversity evaluation metric for machine learning. *Transactions on Machine Learning Research*, 2023. ISSN 2835-8856. URL https://openreview.net/forum?id=g97OHbQyk1.

H. Gong, et al. Integrated mrna sequence optimization using deep learning. *Briefings in Bioinformatics*, 24(1): bbad001, 01 2023. ISSN 1477-4054. doi: 10.1093/bib/bbad001. URL https://doi.org/10.1093/bib/bbad001.

C. R. Harris, et al. Array programming with NumPy. *Nature*, 585:357–362, 2020. doi: 10.1038/s41586-020-2649-2.

M. Heusel, et al. Gans trained by a two time-scale update rule converge to a local nash equilibrium, 2018. URL https://arxiv.org/abs/1706.08500.

C. Hsu, et al. Learning inverse folding from millions of predicted structures. *bioRxiv*, 2022. doi: 10.1101/2022.04.10.487779. URL https://www.biorxiv.org/content/early/2022/09/06/2022.04.10.487779.

J. D. Hunter. Matplotlib: A 2D graphics environment. *Computing in Science & Engineering*, 9(3):90–95, 2007. doi: 10.1109/MCSE.2007.55.

A. Izdebski, et al. Synergistic benefits of joint molecule generation and property prediction, 2025. URL https://arxiv.org/abs/2504.16559.

S. Jayasumana, et al. Rethinking fid: Towards a better evaluation metric for image generation. In *2024 IEEE/CVF Conference on Computer Vision and Pattern Recognition (CVPR)*, pages 9307–9315, 2024. doi: 10.1109/CVPR52733.2024.00889.

M. Kmicikiewicz, et al. Prospero: Active learning for robust protein design beyond wild-type neighborhoods, 2025. URL https://arxiv.org/abs/2505.22494.

T. Kynkäänniemi, et al. Improved precision and recall metric for assessing generative models, 2019. URL https://arxiv.org/abs/1904.06991.

T. J. Lawrence, et al. ampeppy 1.0: a portable and accurate antimicrobial peptide prediction tool. *Bioinformatics*, 37 (14):2058–2060, 11 2020. ISSN 1367-4803. doi: 10.1093/bioinformatics/btaa917. URL https://doi.org/10.1093/bioinformatics/btaa917.

C. Li, et al. Amplify: attentive deep learning model for discovery of novel antimicrobial peptides effective against who priority pathogens. *BMC Genomics*, 23(1):77, Jan 2022. ISSN 1471-2164. doi: 10.1186/s12864-022-08310-4. URL https://doi.org/10.1186/s12864-022-08310-4.

Z. Lin, et al. Language models of protein sequences at the scale of evolution enable accurate structure prediction. *bioRxiv*, 2022.

R. Lorenz, et al. Viennarna package 2.0. *Algorithms for Molecular Biology*, 6(1):26, Nov 2011. ISSN 1748-7188. doi: 10.1186/1748-7188-6-26. URL https://doi.org/10.1186/1748-7188-6-26.

L. McInnes, et al. Umap: Uniform manifold approximation and projection. *The Journal of Open Source Software*, 3 (29):861, 2018.

A. T. Müller, et al. modlamp: Python for antimicrobial peptides. *Bioinformatics*, 33(17):2753–2755, 05 2017. ISSN 1367-4803. doi: 10.1093/bioinformatics/btx285. URL https://doi.org/10.1093/bioinformatics/btx285.

A. Ospanov, et al. Towards a scalable reference-free evaluation of generative models, 2024. URL https://arxiv.org/abs/2407.02961.

F. Pedregosa, et al. Scikit-learn: Machine learning in Python. *Journal of Machine Learning Research*, 12:2825–2830, 2011.

M. Pirtskhalava, et al. Dbaasp v3: database of antimicrobial/cytotoxic activity and structure of peptides as a resource for development of new therapeutics. *Nucleic Acids Research*, 49(D1):D288–D297, 11 2020. ISSN 0305-1048. doi: 10.1093/nar/gkaa991. URL https://doi.org/10.1093/nar/gkaa991.

D. Polykovskiy, et al. Molecular sets (moses): A benchmarking platform for molecular generation models. *Frontiers in Pharmacology*, Volume 11 - 2020, 2020. ISSN 1663-9812. doi: 10.3389/fphar.2020.565644. URL https://www.frontiersin.org/journals/pharmacology/articles/10.3389/fphar.2020.565644.

K. Preuer, et al. Fréchet chemnet distance: A metric for generative models for molecules in drug discovery, 2018. URL https://arxiv.org/abs/1803.09518.

P. F. Rissom, et al. Decoding protein language models: insights from embedding space analysis. *bioRxiv*, 2025. doi: 10.1101/2024.06.21.600139. URL https://www.biorxiv.org/content/early/2025/02/11/2024.06.21.600139.

P. Rodriguez, et al. Lineas: End-to-end learning of activation steering with a distributional loss, 2025. URL https://arxiv.org/abs/2503.10679.

O. Räisä, et al. Position: All current generative fidelity and diversity metrics are flawed, 2025. URL https://arxiv.org/abs/2505.22450.

P. Strashnov, et al. Towards robust evaluation of protein generative models: A systematic analysis of metrics. *bioRxiv*, 2024. doi: 10.1101/2024.10.25.620213. URL https://www.biorxiv.org/content/early/2024/10/29/2024.10.25.620213.

P. Szymczak, et al. Discovering highly potent antimicrobial peptides with deep generative model hydramp. *Nature Communications*, 14(1):1453, Mar 2023. ISSN 2041-1723. doi: 10.1038/s41467-023-36994-z. URL https://doi.org/10.1038/s41467-023-36994-z.

S. Tang, et al. Peptune: De novo generation of therapeutic peptides with multi-objective-guided discrete diffusion, 2025. URL https://arxiv.org/abs/2412.17780.

The pandas development team. pandas-dev/pandas: Pandas. URL https://github.com/pandas-dev/pandas.

M. D. Torres, et al. Generative latent diffusion language modeling yields anti-infective synthetic peptides. *Cell Biomaterials*, 1(9), Oct 2025. ISSN 3050-5623. doi: 10.1016/j.celbio.2025.100183. URL https://doi.org/10.1016/j.celbio.2025.100183.

C. M. Van Oort, et al. Ampgan v2: Machine learning-guided design of antimicrobial peptides. *Journal of Chemical Information and Modeling*, 61(5):2198–2207, May 2021. ISSN 1549-9596. doi: 10.1021/acs.jcim.0c01441. URL https://doi.org/10.1021/acs.jcim.0c01441.

D. Veltri, et al. Deep learning improves antimicrobial peptide recognition. *Bioinformatics*, 34(16):2740–2747, 08 2018. ISSN 1367-4803. doi: 10.1093/bioinformatics/bty179. URL https://doi.org/10.1093/bioinformatics/bty179.

P. Virtanen, et al. SciPy 1.0: Fundamental Algorithms for Scientific Computing in Python. *Nature Methods*, 17: 261–272, 2020. doi: 10.1038/s41592-019-0686-2. URL https://doi.org/10.1038/s41592-019-0686-2.

Y. Wang, et al. A multi-modal contrastive diffusion model for therapeutic peptide generation, 2024. URL https://arxiv.org/abs/2312.15665.

T. Wolf, et al. Transformers: State-of-the-Art Natural Language Processing. pages 38–45. Association for Computational Linguistics, Oct. 2020. URL https://www.aclweb.org/anthology/2020.emnlp-demos.6.

Y. Xiong, et al. mrnabert: advancing mrna sequence design with a universal language model and comprehensive dataset. *Nature Communications*, 16(1):10371, Nov 2025. ISSN 2041-1723. doi: 10.1038/s41467-025-65340-8. URL https://doi.org/10.1038/s41467-025-65340-8.

H. Zhang, et al. Algorithm for optimized mrna design improves stability and immunogenicity. *Nature*, 621 (7978):396–403, Sep 2023. ISSN 1476-4687. doi: 10.1038/s41586-023-06127-z. URL https://doi.org/10.1038/s41586-023-06127-z.

E. Zitzler, et al. Multiobjective evolutionary algorithms: a comparative case study and the strength pareto approach. *IEEE Transactions on Evolutionary Computation*, 3(4):257–271, 1999. doi: 10.1109/4235.797969.
